# Computational identification and binding analysis of orphan human cytochrome P450 4X1 enzyme with substrates

**DOI:** 10.1186/s13104-015-0976-4

**Published:** 2015-01-17

**Authors:** Suresh Kumar

**Affiliations:** Centre for Bioinformatics Research, Institute of Systems Biology (INBIOSIS), University Kebangsaan Malaysia, 43600 UKM Bangi, Selangor Malaysia

**Keywords:** Homology modeling, Human cytochrome, CYP4X1, Molecular docking, Arachidonic acid, Anandamide

## Abstract

**Background:**

Cytochrome P450s (CYPs) are important heme-containing proteins, well known for their monooxygenase reaction. The human cytochrome P450 4X1 (CYP4X1) is categorized as “orphan” CYP because of its unknown function. In recent studies it is found that this enzyme is expressed in neurovascular functions of the brain. Also, various studies have found the expression and activity of orphan human cytochrome P450 4X1 in cancer. It is found to be a potential drug target for cancer therapy. However, three-dimensional structure, the active site topology and substrate specificity of CYP4X1 remain unclear.

**Methods:**

In the present study, the three-dimensional structure of orphan human cytochrome P450 4X1 was generated by homology modeling using Modeller 9v8. The generated structure was accessed for geometrical errors and energy stability using PROCHECK, VERFIY 3D and PROSA. A molecular docking analysis was carried out against substrates arachidonic acid and anandamide and the docked substrates were predicted for drug-likeness, ADME-Tox parameters and biological spectrum activity.

**Results:**

The three-dimensional model of orphan human cytochrome P450 4X1 was generated and assessed with various structural validation programmes. Docking of orphan human cytochrome P450 4X1 with arachidonic acid revealed that TYR 112, ALA 126, ILE 222, ILE 223, THR 312, LEU 315, ALA 316, ASP 319, THR 320, PHE 491 and ILE 492 residues were actively participating in the interaction, while docking of CYP4X1 with anandamide showed that TYR 112, GLN 114, PRO 118, ALA 126, ILE 222, ILE 223, SER 251, LEU 315, ALA 316 and PHE 491 key residues were involved in strong interaction.

**Conclusion:**

From this study, several key residues were identified to be responsible for the binding of arachidonic acid and anandamide with orphan human cytochrome P450 4X1. Both substrates obeyed Lipinski rule of five in drug-likeness test and biological spectrum prediction showed anticarcinogenic activity. Compared to anandamide, arachidonic acid showed strong interaction with cytochrome P450 4X1 and also less health effect in certain human system in ADME-Tox prediction. These findings provide useful information on the biological role and structure-based drug design of orphan human cytochrome P450 4X1.

## Background

Cytochromes P450 are a super family of heme-thiolate proteins which are involved in the oxidative metabolism of both foreign and endogenous compounds [1]. The P450 enzymes are divided into 18 families and 43 subfamilies. The human P450s can also be divided based on their ability to metabolize xenobiotic compounds: sterols, xenobiotics, fatty acids, eicosanoids, and vitamins, and the orphans [2]. About 1/4 of the human P450 enzymes are considered to be “orphans” because, functional information, expression patterns, and regulation are still largely unknown. The orphan enzymes are mostly found in families 1–4, with the largest number found in the P450 4 family [3-5].

CYP4X1 (cytochrome P450, family 4, subfamily X, polypeptide 1) is a protein which in humans is encoded by *CYP4X1* gene and is considered as one of such “orphan” CYPs. The human *CYP4X1* gene is located within the P450 ABXZ gene cluster on chromosome 1. The gene has 12 exons and the predicted protein has 509 amino acids [6]. The tissue distribution of human CYP4X1 is reported to be predominantly found in adult human skeletal muscle, trachea, and aorta [7]. Recent report suggests that CYP4X1 is expressed in brain and in the liver [8,9]. It is involved in drug metabolism and synthesis of cholesterol, steroids and other lipids. Members of the cytochrome P450 4F subfamily are known to primarily oxidize endogenous compounds, for example, fatty acids and arachidonic acid derivatives [10]. The metabolic capabilities of CYP4X1 are largely unknown, yet a recent study has identified arachidonic acid derivatives to have been implicated in a large number of physiologically important processes. A number of P450s, primarily from subfamilies 2C, 2J, 4A and 4F, are known to oxidize arachidonic acid which has been implicated as important signaling mediator. The arachidonic acid derivative anandamide (arachidonoyl ethanol amide) is a natural endocannabinoid found in most human tissues, and acts as an important signaling mediator in neurological, immune and cardiovascular functions. Anandamide (arachidonoyl ethanol amide) has emerged as an important signaling molecule in the neurovascular cascade [11].

Human CYP4X1 amino acid sequence has been identified recently but the three-dimensional structure of this protein is not yet known. Earlier experimental studies of CYP4X1 proposed that arachidonic acid and its derivative anandamide can act as possible substrates [12]. However, to date information on the structure and ligand binding site is not available for CYP4X1. Through homology modeling it is possible to generate realistic model comparable to experimental structures and through docking studies substrate binding energies and important key residues involved in substrate binding can be found. Many Computer-Aided Drug Design (CADD) methodologies have been carried out previously for finding suitable drug target [13-19]. In the present work, three-dimensional model of CYP4X1 was constructed using homology modeling and energy minimization was done to refine the model. After that, arachidonic acid and anandamide were docked into the active sites of the CYP4X1 model. The interaction between CYP4X1 and substrates helped in finding energetically favorable binding sites and the key residues responsible for substrate specificity.

## Methods

### Sequence retrieval

The sequence of human CYP4X1 protein in FASTA format was retrieved from Uniprot Knowledge base (http://www.uniprot.org/) of accession number Q8N118.

### Sequence alignment and homology modeling

For the template selection, PSI-BLAST search was used against Protein Data Bank (PDB) and top ranked six templates (1TQN, 3CZH, 2HI4, 3NA0, 3K9V, 3E4E) were selected for the model building. The templates and target sequence were aligned by using Clustal Omega [20] with default parameters and observed for conserved sequence. Further, the aligned sequence was used as the input to generate homology model of CYP4X1 using Modeller 9v5 [21]. The coordinates for heme were obtained from the template 1TQN and positioned as in the template.

### Energy minimization and structural validation

The constructed CYP4X1 model was further refined by energy minimization using YASARA package [22], and the resulting model was subjected to structural quality assessment. PROCHECK and VERIFY 3D were used for geometric evaluation. The PROSA program was used to assess the energy of residue-residue interaction using a distance-based pair potential and the energy was transformed to a score called Z-score. Residues with negative Z-score indicate reasonable side-chain interactions.

### Binding site analysis

ConSurf was used for identification of the functional regions in the protein. The degree of conservation of the amino acid sites among homologues protein with similar sequences was estimated. The conservation scores were depicted onto the molecular surface of the orphan human cytochrome P450 4X1 to reveal the patches with highly conserved residues that are often important for biological function.

### Ligand optimization

The possible substrates like arachidonic acid and its derivative anandamide were downloaded from the PubChem in Structure Data Format (SDF). Conversion of SDF to Protein Data Bank (PDB) format was carried out using Open Babel program [23]. The MMFF94 force field was used for energy minimization of ligand molecules [24]. Gasteiger partial charges were added to the ligand atoms, non-polar hydrogen atoms were merged and rotatable bonds were defined. Ligand geometries and electric properties were calculated using MOPAC2009 [25].

### Molecular docking

Docking calculations were carried out using DockingServer [26] to compute the free energy of binding on protein model. Essential hydrogen atoms, Kollman united atom type charges and solvation parameters were added with the aid of Auto Dock tools [27]. Affinity (grid) maps of 60X60X60Å grid points and 0.375 Å spacing were generated using the Auto grid program. Auto Dock parameter set and distance dependent dielectric functions were used in the calculation of the Van der Waals and the electrostatic terms respectively. Docking simulations were performed using the Lamarckian Genetic Algorithm (LGA) and the Solis and Wets local search method. Initial position, orientation and torsions of the ligand molecules were set randomly and all rotatable torsions were released during docking. Each docking experiment was derived from 10 different runs that were set to terminate after a maximum of 250000 energy evaluations. During the search population size of 150, translational step of 0.2 Å and quaternion and torsion steps of 5 were applied.

### Prediction of drug-likeness, ADME-Tox and biological spectrum activity

The substrates arachidonic acid and anandamide were subjected drug-likeness prediction using Lipinski rule of five, toxicity prediction using ADME-Tox and also biological activity prediction.

## Results and discussion

### Sequence analysis

**Figure 1:**
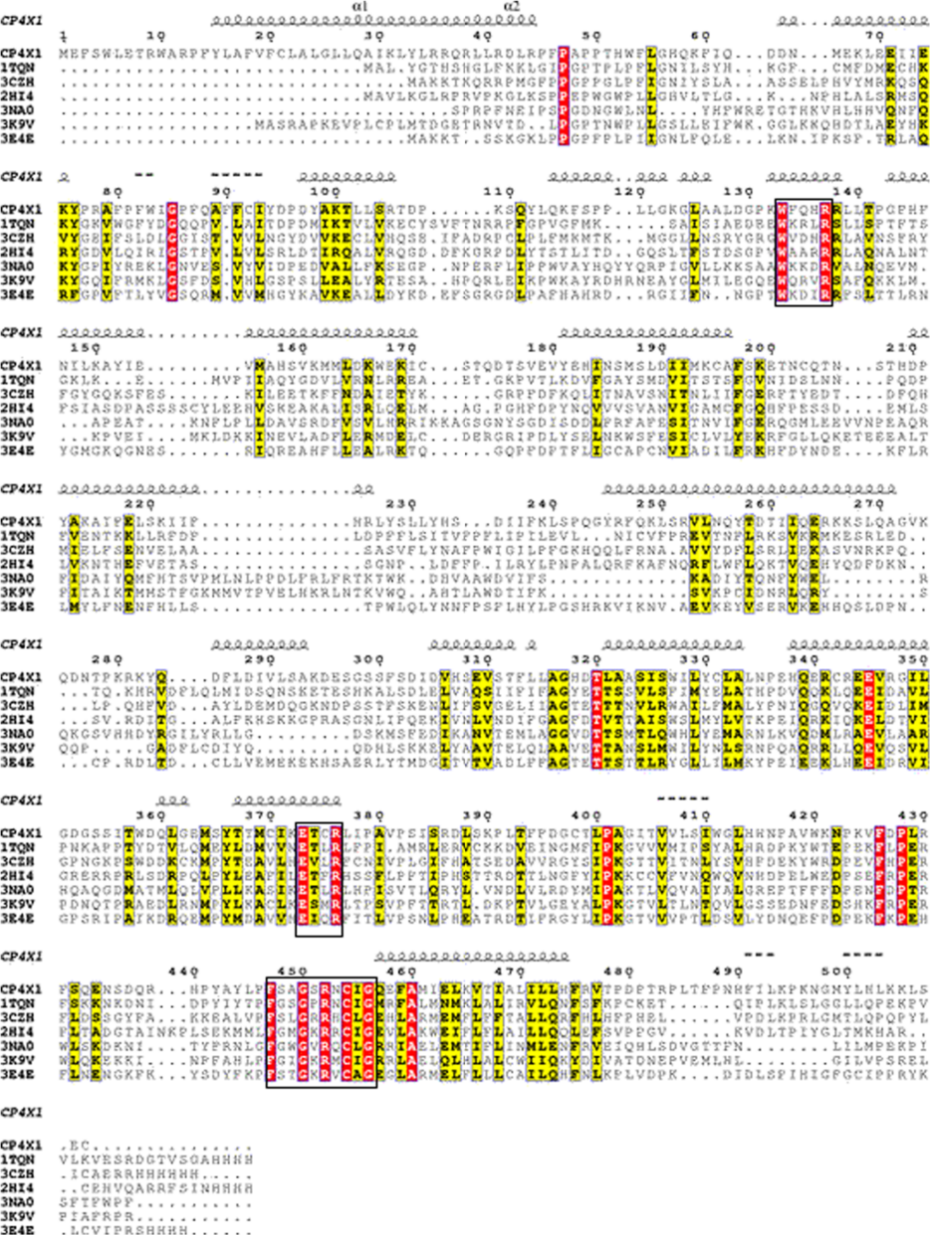
**Multiple alignment of CYP4X1 with templates.** The target CYP4X1 (Q8N118) and six templates (1TQN, 3CZH, 2HI4, 3NA0, 3K9V, 3E4E) were aligned using ClustalO program. The red color indicates conserved residues and yellow colour indicates a semi-consereved substitution. The signature motifs are shown in the box. The alignment figure was prepared using ESpript 2.2.

The amino acid sequence of cytochrome P450 4X1 was retrieved from UniProt database and subjected to trans-membrane prediction using PHD and TMHMM 2.0 servers. The trans-membrane domain was predicted between 13–30 residues. The amino acid sequence of CYP4X1 was queried against Protein Data Bank (PDB) using PSI-Blast with five iterations. PSI-Blast analysis showed that the alignment between target and templates was below 30%. Under this circumstance, use of the multiple templates could provide more reliable structure modeling. Hence, best six templates (1TQN, 3CZH, 2HI4, 3NA0, 3K9V, 3E4E) were selected and the selected template sequence and target sequence (CYP4X1) were subjected to multiple alignments using Clustal Omega with default parameters. The alignment between targets and templates were examined for sequence conservation and signature motifs. The structural motifs were WxxxR in the C helix, ExxR in the K helix and essential one FxxGxxxCxG in L helix. Characteristic motif for the CYP super family, which includes conserved cystine residue that ligates to the Fe of the heme was observed (Figure 1). Several signature motifs were conserved for CYP4X1 which is in agreement with previous findings [15,28]. The conservation of sequence and signature motifs elucidates that model based on this alignment is reliable. In the available template, the first 20 residues of the N-terminal were deleted in order to facilitate its crystallization. Since the N-terminus does not affect the substrate binding, the corresponding first 20 residues were not modeled in the CYP4X1 model.

### Structure analysis

**Figure 2:**
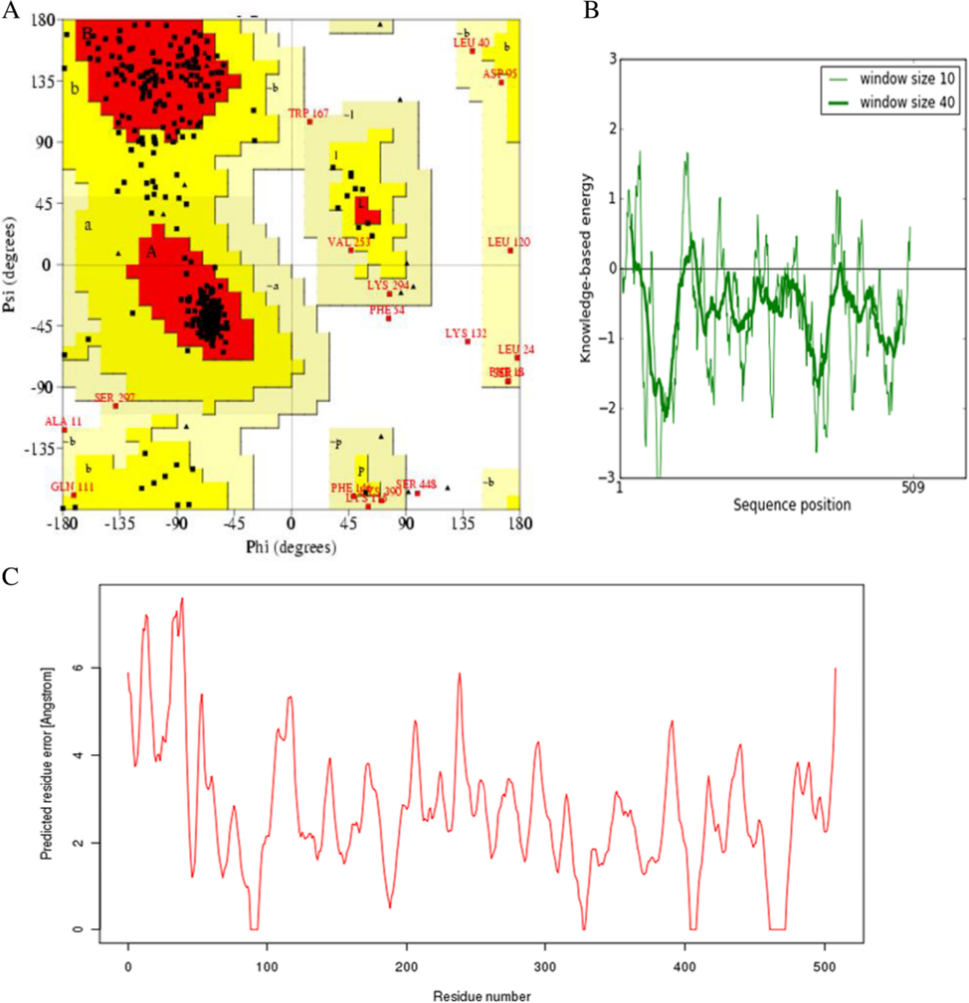
**Structural Quality Assessment. (A)** Ramachandran plot of orphan cytochrome P450 4X1 protein showing the distribution of residues in favored, allowed and outlier regions. **(B)** PROSA Z-score with respect to residue. **(C)** Q-Mean score.

The alignment between templates and target sequence were submitted to Modeller for the construction of homology model of CYP4X1. The loops were modeled through the server ModLoop and subjected to energy minimization using YASARA package. Energy minimization was performed by YAMBER force field implemented in YASARA package to get optimized model structure having the initial energy of 72759.7 kJ/mol to a final energy of −247540.1 kJ/mol. The energy minimized homology model of CYP4X1 was subjected to several structural validation programs like PROCHECK, VERIFY 3D, QMEAN, PROSA, and PROVE. The PROCHECK [29] analysis based on Ramachandran plot provides an idea on the stereo chemical quality of the protein model. It highlights regions of the proteins which appear to have unusual geometry and provides an overall assessment of the structure. The PROCHECK evaluation showed the residues in most favored regions as 81.9%, residues in additional allowed regions as 14.8%, residues in generously allowed regions as 2.6% and residues in disallowed regions as 0.7% (Figure 2A). For a good quality model, the residues located in the core and allowed regions should be over 90% which is the case for the model presented here (since 81.4% + 14.8% = 96.2%). In order to check the native protein folding energy of the model, PROSA II [30] analysis was made and mean force potentials and averaged over all residues in the structure were determined. The PROSA II analysis showed Z-score of −8.33 (Figure 2B), and PROVE [31] analysis showed average Z-score of 0.97 and Z-score RMS value of 23.24. QMEAN [32] is a scoring function of a linear combination of six structural descriptors with the score ranging between 0 and 1 and higher value reflecting a better quality of input model. The QMEAN score of the present model is 0.517 (Figure 2C). From the results of entire structural validation program it is inferred that the homology modeled protein is reliable for further study.

**Figure 3:**
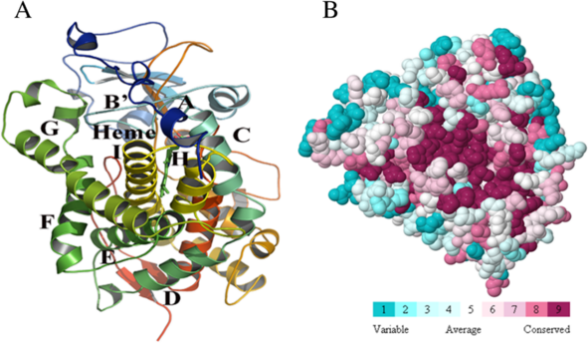
**Homology model and functional region prediction of CYP4X1. (A)** Ribbon schematic representations of the homology model of CYP4X1. Major helices are labeled. **(B)** Identification functional region of CYP4X1 using ConSurf. The colour-coding bar shows the colouring scheme. Conserved amino acids are coloured bordeaux, residues with average conservation by white, and variable amino acids by turquoise.

The final model was deposited in Protein Model Database (PMDB) [33] and it is available under ID: PM0079202. The overall topology of homology model of CYP4X1 consists of twelve helices, namely, A-L, five antiparallel beta sheets and their connecting loops. The heme is positioned between two structurally conserved helices, I and L (Figure 3A). Highly conserved theronine residue is located in the middle of helix I as seen in many available mammalian CYP structures. This conserved residue has been suggested to participate in the proton delivery and play an important role in the dioxygen bond cleavage during catalytic cycle [34].

### Binding site prediction

ConSurf [35] was used to characterize the functional regions in the protein. It identifies by considering the degree of conservation of the amino acid sites among their sequence homologues. The conservation grades were projected on the molecular surface of the CYP4X1 protein to reveal the patches with highly conserved residues that are often important for biological function. The surface residues with the most conserved amino acids are shown colored in bordeaux, residues with average conservation in white and variable amino acids in turquoise in the protein structure in Figure 3B.

Predicting ligand binding sites can reduce the conformational space of docking and also can provide insights into their molecular functions.

### Molecular docking

**Figure 4:**
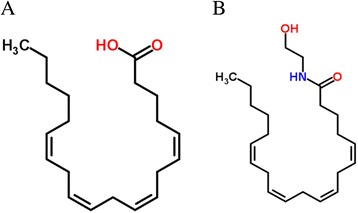
**2D structures of substrates. (A)** Arachidonic acid. **(B)** Anandamide.

**Figure 5:**
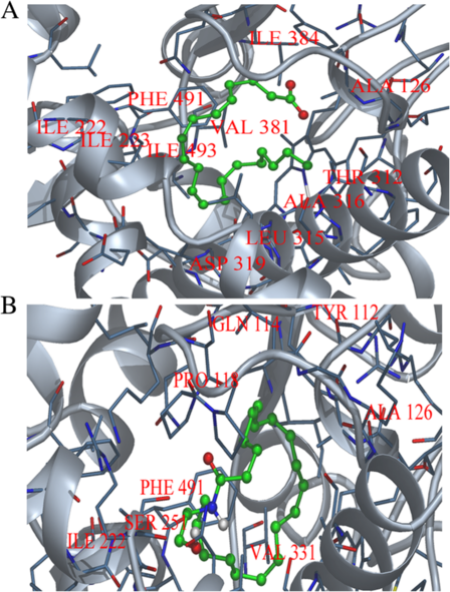
**Interaction of CYP4X1 with substrates. (A)** Arachidonic acid and **(B)** Anandamide. Key residues are labeled. Substrates are shown in green stick.

In order to understand enzyme-substrate interaction and to determine the key residues responsible for interaction, the model of CYP4X1 was docked with selected substrates using DockingServer. DockingServer integrates a number of popular software like Auto Dock and MOPAC for accurate ligand geometry optimization, energy minimization, charge calculation, docking calculation and protein-ligand complex representation. The calculated free energy of binding of CYP4X1 with arachidonic acid (Figure 4A) and anandamide (Figure 4B) were −7.76 and −5.76 kcal/mol respectively (Table 1). The negative and low value of free energy of binding indicates a strong favorable bond between CYP4X1 and arachidonic acid in most favorable conformations. Docking of CYP4X1 with arachidonic acid revealed that major residues involved were TYR 112, ALA 126, ILE 222, ILE 223, THR 312, LEU 315, ALA 316, ASP 319, THR 320, PHE 491 and ILE 492 (Figure 5A). With anandamide, major residues were TYR 112, GLN 114, PRO 118, ALA 126, ILE 222, ILE 223, SER 251, LEU 315, ALA 316 and PHE 491 (Figure 5B). The common residues in both arachidonic acid and anandamide were TYR 112, ILE 223, LEU 315, ALA 316 and PHE 491 (Table 2).

**Table 1:** **Docking of CYP4X1 with selected ligands**

**Ligand**	**Free energy of binding** **(kcal** **/mol)**	**Inhibition constant**, **Ki** **(uM)**	**vdW** **+** **Hbond** **+** **desolv energy** **(kcal** **/mol)**	**Electrostatic energy** **(kcal** **/mol)**	**Total inter molec. energy** **(kcal/** **mol)**	**Interact. surface**
Arachidonic acid	−7.76	2.05	−10.87	+0.03	−10.84	813.954
Anandamide	−5.76	59.98	−10.53	−0.03	−10.57	1042.601

**Table 2:** **Details of intermolecular interactions in the binding site of orphan human cytochrome P450 4X1**

**Ligand complex**	**Hydrophobic**	**Polar**	**Other**
Arachidonic acid	O1 [3.81]- GLN114	C4 [3.87] - TYR112	C8 [3.80]- GLN114
		C17 [2.99] - ILE223	C13 [3.35]- GLN114
		C14 [3.51] - ILE223	C18 [3.77] - GLN114
		C19 [3.80] - ILE223	C4 [3.83] - GLN114
		C12 [3.29] - PHE247	O2 [3.49]- ALA126
		C15 [3.89] - PHE247	O2 [3.53] - LEU315
		C17 [3.40] - LEU315	C6 [3.78] - SER385
		C14 [3.27]- LEU315	
		C10 [3.53] - LEU315	
		C11[3.43] - LEU315	
		C20 [3.60]-LEU315	
		C19 [3.41] - PHE491	
		C16 [3.69]-PHE491	
		C6 [3.40] - PHE491	
		C9 [3.70] - PHE491	
		C5 [3.70] - PHE491	
		C2 [3.38] – PHE491	
		C4 [3.51] – PHE491	
Anandamide	C16 [3.25] -TYR112		C16 [3.86] -TYR112
	C1 [3.43] -LEU121		C13 [3.51]-GLN114
	C8 [3.70] -LEU121		C10 [3.89] -GLN114
	C3 [3.89] - LEU121		C9 [3.61]-GLN114
	C2 [3.81] -LEU121		C21 [3.24] -GLN114
	C4 [3.43] -LEU121		C19 [3.09] -GLN114
	C1 [3.78] -ALA126		O1 [3.24]- ALA126
	C4 [3.48] -ALA126		O2 [3.36]- PHE491
	C18 [3.80] - ILE222		
	C20 [3.76] - ILE222		
	C14 [3.73] -ILE223		
	C11 [3.84] - ILE223		
	C20 [3.56] - HIS225		
	C17 [3.66] - HIS225		
	C10 [3.75] - PHE247		
	C3 [3.54]- LEU315		
	C5 [3.83] -ALA316		
	C7 [3.29] - ALA316		
	C19 [3.34] -PHE491		
	C21 [3.48] -PHE491		
	C14 [3.51] -PHE491		
	C11 [3.70] - PHE491		
	C15 [3.85] -PHE491		
	C22 [3.76] -PHE491		

### Evaluation of drug-likeness and ADME-Tox

Both arachidonic acid and anandamide were evaluated for drug-likeness using Lipinski rule of five [36]. The ‘rule of five’ imposes limitation on the logP (the logarithm of octanol/water partition coefficient), molecular weight and the number of hydrogen bond acceptors and donors. The rule states that most ‘drug-like’ molecules have logP <5, molecular weight <500, number of hydrogen bond acceptors <10 and number of hydrogen bond donors <5. Molecules violating more than one of these rules may have problems with bioavailability. Both arachidonic acid and anandamide do not violate the Lipinski rule of five parameters to be an orally active compound (Table 3). Moreover, according to Freitas [37], the oral bioavailability is inversely proportional to topological polar surface area (TPSA). In this study, arachidonic acid has lower topological surface area value than anandamide suggesting arachidonic acid has better oral bioavailability than anandamide. Analysis of pharmacological parameters using ADME-Tox evaluation revealed that both arachidonic acid and anandamide have comparable values on specific parameters related to absorption, distribution, metabolism, excretion and toxicity. But arachidonic acid has less health effects on human than anandamide (Table 4).

**Table 3:** **Ligand Properties**

**Ligand**	**Molar weight**	**LogP**	**H Acceptor**	**H Donor**	**pKa**	**pKb**	**logD**	**logK**	**TPSA**
Arachidonic acid	303.46	6.16	2	1	4.82	0	3.99	−19.64	37.3
Anandamide	347.54	4.80	2	2	15.68	−0.39	4.80	−33.58	49.33

**Table 4:** **ADME**-**Tox prediction**

**ADME**-**Tox**	**Arachidonic acid**	**Anandamide**
Solubility^a^ H2O (mg mL^−1^)	0.01	0.55
LogD^b^ pH 1.7 (stomach)	6.75	6.18
LogD^b^ pH 4.6 (duodenum)	6.57	6.18
LogD^b^ pH 6.5 (jejunum, ileum)	5.14	6.18
LogD^b^ pH 7.4 (blood)	4.26	6.18
LogD^b^ pH 8.0 (colon)	3.69	6.18
% oral bioavailability^c^	>30	>30
Absorption^d^ (cm s^−1^)	7.51x10^−4^	7.2x10^−4^
Distribution^e^ (L kg^−1^)	0.5	3.96
Ames test^f^	0.84	0.84
Prob. of Blood effect^g^	0.47	0.52
Prob. of Cardiovascular system effect^g^	0.07	0.30
Prob. of gastrointestinal system effect^g^	0.04	0.07
Prob. of kidney effect^g^	0.69	0.75
Prob. of liver effect^g^	0.33	0.32
Prob. of lung effect^g^	0.79	0.70

^a^Calculates compound’s solubility in a buffer at a specified pH value.

^b^Calculates logarithm of the apparent octanol–water partition coefficient D at various pH.

^c^Estimates the probability of a compounds bioavailability being above 30 and 70%.

^d^Estimates human jejunum permeability.

^e^Calculates the apparent volume of distribution for a compound in L kg-1.

^f^Test for assessing mutagenic properties of the compounds.

^g^Estimates probability of blood, gastrointestinal system, kidney, liver and lung effect at therapeutic dose range.

### Biological spectrum predictions

Computer software PASS [38,39] predicts simultaneously several hundreds of biological activities depending upon the chemical structures of compounds such as the predicted activity spectrum giving probable activity (Pa) and probable inactivity (Pi). Prediction of this spectrum by PASS is based on SAR analysis of the training set containing more than 35,000 compounds, which correlates with more than 500 kinds of biological activities. Pa and Pi values are independent and their values can vary from 0 to 1.

The more the Pa value, the less is the probability of false positives in the set of compounds selected for biological testing. For example, if one selects compounds for which a particular activity has Pa ≥ 0.9, the expected probability to find inactive compounds in the selected set is very low, but about 90% of active compounds are missed. If compounds with Pa ≥ 0.8 are chosen, the probability to find inactive compounds is also low, but about 80% of active compounds are missed. By default, in PASS, value with Pa = Pi is chosen as a threshold and all compounds with Pa > Pi are suggested to be active. Another criterion for selection is the novelty of compounds. If, Pa value is high, sometimes one may find close analogues of known biologically active substances among the tested compounds. For example, if, Pa > 0.7, the chance to find the activity in experiment is high, but in some cases the compound may occur to be the close analogue of known pharmaceutical agents. If, 0.5 < Pa < 0.7 the chance to find the activity in experiment is less, but the compound is not so similar to the known pharmaceutical agents. If, Pa < 0.5 the chance to find the activity in experiment is even less, but if it is confirmed the compound may occur to be a new chemical entity. Results of prediction of the biological spectrum of substrates estimated when Pa > 0.5 is shown in Table 5. Both the substrates show similar biological spectrum for anticarcinogenic activity.

**Table 5:** **Biological spectrum predictions of arachidonic acid and anandamide**

**S.No**	**Pa**	**Pi**	**Activities**
1	0.784	0.020	Mucositis treatment
2	0.712	0.004	Antimutagenic
3	0.670	0.035	Antiviral
4	0.662	0.005	Antipyretic
5	0.623	0.099	Urologic disorders treatment
6	0.598	0.073	Antineoplastic (non-small cell lung cancer)
7	0.593	0.052	Antacid
8	0.584	0.054	Digestive functional disorders treatment
9	0.578	0.009	Antihelmintic (Nematodes)
10	0.562	0.051	Antineoplastic (gastric cancer)
11	0.556	0.115	Neuroprotector
12	0.555	0.004	Antiuremic
13	0.543	0.009	Acaricide
14	0.538	0.080	Antineurotoxic
15	0.536	0.117	Antineoplastic (head/neck cancer)
16	0.536	0.032	Anti-inflammatory, intestinal
17	0.526	0.038	Allergic conjunctivitis treatment
18	0.524	0.004	Antispirochetal
19	0.518	0.028	Cytoprotectant
20	0.512	0.008	Anticarcinogenic

## Conclusion

The orphan human cytochrome P450 4X1 has been suggested to be a potential drug target for cancer therapy. Lack of the structural information about this enzyme hinders the detailed characterization of its biological functions and its applications in structure based design. For this reason, the three-dimensional model of orphan human cytochrome P450 4X1 was constructed using homology modeling. To provide useful information to characterize the enzyme’s function, two known substrates, arachidonic acid and anandamide were docked into the active sites and then refined by energy minimization to determine favorable binding modes. Several key residues TYR 112, ILE 223, LEU 315, ALA 316 and PHE 491 were identified to have involved in binding of arachidonic acid and anandamide. These key residues are expected to affect the catalytic activity and can be used as candidates for further mutagenesis studies. Both the substrates does not violate Lipinski rule of five in drug-likeness test, while in ADME-Tox prediction, arachidonic acid shows less health effects on cardiovascular system, gastrointestinal system and kidney of human when compared to anandamide. In biological activity spectrum predictions, both arachidonic acid and anandamide show anticarcinogenic activity. However, further *in*-*vivo* validation and conformation of the present finding is required. The results of this study will be useful for structure-based drug design of orphan human cytochrome P450 4X1.

## References

[1] Danielson P (2002). The cytochrome P450 superfamily: biochemistry, evolution and drug metabolism in humans. Curr Drug Metab.

[2] Stark K, Guengerich FP (2007). Characterization of orphan human cytochromes P450. Drug Metab Rev.

[3] Guengerich FP, Cheng Q (2011). Orphans in the human cytochrome P450 superfamily: approaches to discovering functions and relevance in pharmacology. Pharmacol Rev.

[4] Guengerich FP, Wu Z-L, Bartleson CJ (2005). Function of human cytochrome P450s: characterization of the orphans. Biochem Biophys Res Commun.

[5] Guengerich FP, Tang Z, Salamanca-Pinzón SG, Cheng Q (2010). Characterizing proteins of unknown function: orphan cytochrome P450 enzymes as a paradigm. Mol Interv.

[6] Hsu M-H, Savas Ü, Griffin KJ, Johnson EF (2007). Human cytochrome p450 family 4 enzymes: function, genetic variation and regulation. Drug Metab Rev.

[7] Savas Ü, Hsu M-H, Griffin KJ, Bell DR, Johnson EF (2005). Conditional regulation of the human CYP4X1 and CYP4Z1 genes. Arch Biochem Biophys.

[8] Bylund J, Zhang C, Harder DR (2002). Identification of a novel cytochrome P450, CYP4X1, with unique localization specific to the brain. Biochem Biophys Res Commun.

[9] Choudhary D, Jansson I, Stoilov I, Sarfarazi M, Schenkman JB (2005). Expression patterns of mouse and human CYP orthologs (families 1–4) during development and in different adult tissues. Arch Biochem Biophys.

[10] Capdevila JH, Falck JR (2002). Biochemical and molecular properties of the cytochrome P450 arachidonic acid monooxygenases. Prostaglandins Other Lipid Mediat.

[11] Battista N, Fezza F, Maccarrone M (2004). Endocannabinoids and their involvement in the neurovascular system. Curr Neurovasc Res.

[12] Stark K, Dostalek M, Guengerich F (2008). Expression and purification of orphan cytochrome P450 4X1 and oxidation of anandamide. FEBS J.

[13] Lewis DF, Ito Y (2008). Human cytochromes P450 in the metabolism of drugs: new molecular models of enzyme-substrate interactions. Expert Opin Drug Metab Toxicol.

[14] Kumar S (2011). Molecular modeling and identification of substrate binding site of orphan human cytochrome P450 4F22. Bioinformation.

[15] Kumar S (2011). Comparative modeling and molecular docking of orphan human CYP4V2 protein with fatty acid substrates: Insights into substrate specificity. Bioinformation.

[16] Alonso H, Bliznyuk AA, Gready JE (2006). Combining docking and molecular dynamic simulations in drug design. Med Res Rev.

[17] Wang Y-T, Chan C-h, Su Z-Y, Chen C-L (2010). Homology modeling, docking, and molecular dynamics reveal HR1039 as a potent inhibitor of 2009 A (H1N1) influenza neuraminidase. Biophys Chem.

[18] Ian I-F, Luis R-TJ, Pablo C-VJ, Luis V-SJ, Normande C-I, Beatriz Z-L (2013). Identification of pharmacological targets combining docking and molecular dynamics simulations. Am J Agric Biol Sci.

[19] Tambunan USF, Pratomo H, Parikesit AA (2013). Modification of Kampmann A5 as potential fusion inhibitor of dengue virus using molecular docking and molecular dynamics approach. J Med Sci.

[20] 20. Sievers F, Wilm A, Dineen D, Gibson TJ, Karplus K, Li W, Lopez R, McWilliam H, Remmert M, Söding J: Fast, scalable generation of high-quality protein multiple sequence alignments using Clustal Omega. *Molecular systems biology* 2011, 7(1) 10.1038/msb.2011.75 PMC3261699 21988835

[21] Šali A, Blundell TL (1993). Comparative protein modelling by satisfaction of spatial restraints. J Mol Biol.

[22] Krieger E, Koraimann G, Vriend G (2002). Increasing the precision of comparative models with YASARA NOVA—a self‐parameterizing force field. Proteins.

[23] O’Boyle NM, Banck M, James CA, Morley C, Vandermeersch T, Hutchison GR (2011). Open Babel: an open chemical toolbox. J Cheminform.

[24] Halgren TA (1996). Merck molecular force field. II. MMFF94 van der Waals and electrostatic parameters for intermolecular interactions. J Comput Chem.

[25] Stewart JJ (1990). MOPAC: a semiempirical molecular orbital program. J Comput Aided Mol Des.

[26] Bikadi Z, Hazai E (2009). Application of the PM6 semi-empirical method to modeling proteins enhances docking accuracy of AutoDock. J Cheminform.

[27] Morris GM, Huey R, Lindstrom W, Sanner MF, Belew RK, Goodsell DS (2009). AutoDock4 and AutoDockTools4: Automated docking with selective receptor flexibility. J Comput Chem.

[28] Lewis D, Watson E, Lake B (1998). Evolution of the cytochrome < i > P</i > 450 superfamily: sequence alignments and pharmacogenetics. Mutat Res.

[29] Laskowski RA, MacArthur MW, Moss DS, Thornton JM (1993). PROCHECK: a program to check the stereochemical quality of protein structures. J Appl Crystallogr.

[30] Sippl MJ (1993). Recognition of errors in three‐dimensional structures of proteins. Proteins.

[31] Pontius J, Richelle J, Wodak SJ (1996). Deviations from standard atomic volumes as a quality measure for protein crystal structures. J Mol Biol.

[32] Benkert P, Tosatto SC, Schomburg D (2008). QMEAN: a comprehensive scoring function for model quality assessment. Proteins.

[33] Castrignanò T, De Meo PDO, Cozzetto D, Talamo IG, Tramontano A (2006). The PMDB protein model database. Nucleic Acids Res.

[34] Denisov IG, Makris TM, Sligar SG, Schlichting I (2005). Structure and chemistry of cytochrome P450. Chem Rev.

[35] Glaser F, Pupko T, Paz I, Bell RE, Bechor-Shental D, Martz E (2003). ConSurf: identification of functional regions in proteins by surface-mapping of phylogenetic information. Bioinformatics.

[36] Lipinski CA (2004). Lead-and drug-like compounds: the rule-of-five revolution. Drug Discov Today Technol.

[37] Freitas MP (2006). MIA-QSAR modelling of anti-HIV-1 activities of some 2-amino-6-arylsulfonylbenzonitriles and their thio and sulfinyl congeners. Org Biomol Chem.

[38] Anzali S, Barnickel G, Cezanne B, Krug M, Filimonov D, Poroikov V (2001). Discriminating between drugs and nondrugs by prediction of activity spectra for substances (PASS). J Med Chem.

[39] Lagunin A, Stepanchikova A, Filimonov D, Poroikov V (2000). PASS: prediction of activity spectra for biologically active substances. Bioinformatics.

